# Thrombocytopenia is not mandatory to diagnose haemolytic and uremic syndrome

**DOI:** 10.1186/1471-2369-14-3

**Published:** 2013-01-08

**Authors:** Marion Sallée, Khalil Ismail, Fadi Fakhouri, Henri Vacher-Coponat, Julie Moussi-Francés, Véronique Frémaux-Bacchi, Stéphane Burtey

**Affiliations:** 1 APHM, Hopital de la Conception, centre de néphrologie et transplantation rénale, Aix-Marseille Univ, Marseille, 13005, France; 2 Département de Nephrologie, INSERM UMR643, CHU de Nantes, 1 place Alexis-Ricordeau, Nantes, 44000, France; 3 Service d’immunologie biologique, hôpital Européen Georges-Pompidou, Assistance publique–Hôpitaux de Paris, 20-40, rue Leblanc, Paris cedex 15, 75908, France

**Keywords:** Hemolytic and uremic syndrome, Thrombocytopenia, Complement

## Abstract

**Background:**

Hemolytic and uremic syndrome (HUS) diagnosis involves association of non immune hemolytic anemia, thrombocytopenia, and renal failure. HUS without thrombocytopenia has been observed, we call it partial HUS. Its real frequency and outcome are unknown. The aim of this study was to determine the prevalence of patients with normal platelets count in two HUS cohorts and to compare their outcome to patients with thrombocytopenia.

**Methods:**

We retrospectively identified HUS diagnosis in two different cohorts. The first cohort was from a single center and consisted of all cases of HUS whatever the aetiology, the second was multicentric and consisted of atypical HUS patients. These cohorts were divided into two groups depending on the presence or absence of thrombocytopenia. Clinical and biological data were compared between thrombopenic and non thrombopenic group.

**Results:**

We identified 13% (20/150) of patients with normal platelets count: 10 episodes (18%) of HUS in six patients (14%) in the monocentric cohort and 14 patients (13%) with 17 episodes (12%) in the multicentric cohort of atypical HUS. Groups differed in platelets count and LDH level. In both cohorts, renal outcome was similar to patient presenting with thrombocytopenia.

**Conclusion:**

HUS with normal platelets count is not infrequent. Relative to classical clinical presentation of HUS, partial HUS has similar characteristics and identical poor renal outcome and so must be treated in the same way.

## Background

Thrombotic microangiopathy (TMA) is defined as a typical histology characterized by intimal proliferation and/or endothelial swelling with luminal fibrin deposition in arterial or capillary beds. In the kidney, mesangiolysis, red-cell fragments and double contours of capillary basement can also be found in TMA [1]. TMA is typically associated with non-immune mechanical haemolytic anaemia, thrombocytopenia and organ dysfunction.

Two major syndromes can be distinguished: the haemolytic uremic syndrome (HUS) and the thrombotic thrombocytopenic purpura (TTP) depending on, in general speaking, the main organ function altered, kidney in HUS and brain in TTP. Differences in pathogenicity are observed. TTP is associated with a defective function of the von Willebrand factor cleaving protease (ADAMTS13) [2,3]. HUS typically results from food poisoning with enterohemorrhagic strains of *Escherichia coli* or other bacteria producing a shiga-like toxin (STEC-HUS). Mutations in *CFH*, *CFI*, *CFB*, *MCP*, and *C3* predispose to atypical HUS (aHUS) [4-10]. Secondary causes of HUS, due to variety of causes (such as drugs, malignancy, systemic disease…) occur not only in adults but also in childhood.

Partial HUS (normal platelet count) is not well characterized; we believed that this can lead to misdiagnosis of HUS and as a result, undertreated patients or at least delayed therapy. This delay could reduce survival.

If partial HUS is a known entity, its real frequency is unknown. In literature, few studies address this fact [11,12]. An occurrence between 0% and 44% is reported. Therefore we conducted a study to describe the real prevalence of subjects with normal platelets count in a single nephrology centre adult cohort of HUS, reflecting the whole spectrum of HUS. In order to confirm prevalence of this entity, we also analyzed a multicentre French cohort of adults with aHUS.

## Methods

We conducted a retrospective study in the nephrology centre at Hôpital La Conception (AP-HM, Marseille, France). Using a computerized database, we identified all patients with a HUS diagnosis admitted between January 2000 and June 2010. All patients’ medical records were reviewed. Relevant clinical and biological data were collected. Children were excluded.

Partial HUS was suspected by the association of

a mechanical (with schizocytes on blood smear), non immune (negative coombs test) haemolytic anaemia (haemoglobin under 12 g/dl, haptoglobin under 0,4 g/l and lactate deshydrogenase (LDH) >250 UI/L)

and acute renal failure (blood creatinine >120 μmol/l).

To confirm the diagnosis of partial HUS, patients were required to have either biopsy proven TMA, and/or mutation in genes coding for proteins involved in the complement alternative pathway, and/or exposition to a drug associated with HUS like gemtacibine and/or recurrence of partial HUS associated with response to plasmatherapy.

Complete HUS was diagnosed by the association of a mechanical, non immune haemolytic anaemia, thrombocytopenia (platelets count below 150 G/L) and acute renal failure.

TTP was excluded by ADAMTS 13 activity above 20%.

We retrospectively compared the group of patients with thrombocytopenia with those with normal platelets count at diagnosis. Treatments before hospital admission, clinical and biological data on admission, treatments administered, clinical outcome, renal outcome and the number of relapses were collected. In cases of relapses, we noted whether the diagnostic criteria were the same.

Platelets counts were taken on the admission. However partial HUS group was defined by the fact that during all the disease, platelets count never decrease below 150 G/L.

Data from the thrombocytopenic group and from the partial HUS group were analyzed further by Fisher’s exact tests and Mann–Whitney test (two tailed) analyses.

To confirm our main result, we used a multicentre French database to identify adult with aHUS referred between 2000 and 2008 to the laboratory of immunology at Hôpital Européen Georges Pompidou (Paris, France), the French reference centre for the evaluation of complement disorders in human diseases. Diagnostic criteria used were the same as those used for the first cohort. Children were excluded. Nine patients included in the first cohort were excluded. Clinical and biological data were collected by the physicians who referred the patients. So, while the Marseilles cohort included all coming HUS patients regardless of the cause, the multicentre French cohort was most restrictive, limited to aHUS.

This study is in compliance with the Helsinki Declaration. We did not need the approval of an ethic committee to access and use the data. The access to hospital files was given by the head of the department of nephrology in Marseilles and the head of the department of immunology for the French cohort.

## Results

In the Marseille database, we identified 43 patients, presenting 56 episodes of HUS, over the course of ten years. Six of the 43 patients (14%) had a total of 10 episodes (10/56, 18%) of partial HUS.

Patients’ characteristics are summarized in Table 1. Patients were young (median age: 45 years [25–64]) with a majority of females (60%). Aetiologies of HUS were those commonly described in HUS such as toxic causes, mutations in proteins involved in alternative pathway of the complement, and post infectious diseases.

**Table 1:** Data from the Marseille cohort

	**All patients**	**Patients with normal platelets count**	**Patients with thrombocytopenia**	**p**
*Number of patients*	43	6	37	
*Number of episodes*	56	10	46	
*Number of relapsing patients*	10	3	7	0.88
*Sex (%male)*	40%	17%	43%	0.40
*Age (years)*	45 [25–64]	40 [32–50]	44 [23–66]	0.78
*Biopsy proven TMA*	10 (23%)	3 (50%)	7 (19%)	0.38
***Etiology identification***	**24 (56%)**	**3 (50%)**	**21 (57%)**	
*Identified mutation*	6	**2**	4	
*Anti-factor H Autoantibody*	1		1	
*Toxic*	5	**1**	4	
*Cancer associated*	2		2	
*Graft associated*	2		1	
*Autoimmune disease*	3		3	
*Shiga like toxin*	4		4	
*Post infectious disease*	2		2	
***Potential explanation***	**10 (23%)**	**2 (33%)**	**8 (22.5%)**	
*Familial form*	2		2	
*Recurrent form*	4	2	2	
*Associated with low C3*	4		4	
***Idiopathic***	**9 (21%)**	**1 (17%)**	**8 (22.5%)**	
*Pregnancy associated HUS*	3 (11,5%)	2 (40%)	1 (5%)	0.151
Platelets (G/L)*	66 [55–108]	234 [183–265]	53 [27–88]	< 0.001
*Hb (g/dl)*	7.6 [7.1-8.9]	8 [7.2-9.8]	7.7 [7.1-8.8]	0.38
LDH* (UI/L)	1012 [607–202]	612 [400–705]	1074 [659–2192]	0.012
*Haptoglobin*	0 [0–0]	0 [0–0,28]	0 [0–0]	0.064
Schizocytes %*	2.2 [1.5-3.8]	0.8 [0–2]	3 [1.5-4.4]	< 0.001
*Creatinine (mg/dl)*	3.4 [2.4-5.5]	2.5 [1.4-5.3]	3.5 [2.5-5.6]	0.76
*Proteinuria (g/24h)*	2.2 [1.1 – 3.6]	1.5 [0.8 – 2.3]	2.32 [1.2 – 4]	0.15
*HD at the diagnosis*	12 (21%)	2 (20%)	10 (22%)	0.68
*HD during the episode*	34 (61%)	5 (50%)	29 (63%)	0,6
*Death (%)*	1 (2%)	1 (17%)	0 (0%)	0,6
*Plasmatherapy*				
*Number of episodes treated*	52 (93%)	8 (80%)	44 (96%)	0.88
*Number of plasmatherapy sessions*	15 [12–18]	15 [6–16.5]	14 [12–20]	

For Numeric value, data are express in Median value [25%-75%], for categories, data are express in number of observation (%). Test used, Mann Whitney test (quantitative data) and Fisher's test for proportion and rate. *Note,* Conversion factors for units, serum creatinine in mg/dL to mol/L, ×88.4; serum urea nitrogen in mg/dL to mmol/L.

All patients exhibited an acute renal failure (median plasma creatinine level: 3.4 mg/dl [2.4-5.5]) and mechanical, non immune haemolytic anaemia (median Hb: 7.6 g/dl [7.1-8.9], median LDH: 1012 UI/L [607–202], and haptoglobin was not measurable). No difference was observed for creatinine level, haemoglobin level and proteinuria between the thrombocytopenic and the partial HUS groups, while LDH levels and schizocytes count were higher in the thrombocytopenic group.

Relapses were described in 7 patients (7/37, 19%) in the thrombocytopenic group and in 3 patients (3/6, 50%) in the partial HUS group. Platelets count (<150 G/L or ≥ 150 G/L) during relapses were similar to that at first episode.

Plasmatherapy were used for both groups with no differences between number of treated patient and number of plasmatherapy session for an episode.

Table 2 summarized patients’ characteristics for the French cohort. We identified 107 patients presenting 145 episodes of HUS. Fourteen of the 107 patients (13%) presented 17 episodes (17/145, 12%) of partial aHUS. Aetiologies in this cohort represent a large panel of mutations in proteins implicated in complement alternative pathway or a familial history of HUS. As in the Marseille cohort, the LDH levels were significantly higher in the thrombocytopenic aHUS group compared to the partial aHUS group. For all other variables studied, no difference was observed.

**Table 2:** Data from the multicentric French cohort

	**All patients**	**Patients with normal platelets count**	**Patients with thrombocytopenia**	**P**
*Number of patients*	107	14	93	
*Number of episodes*	145	17	128	
*Number of relapsing patients*	18 (20%)	2 (14%)	19 (20%)	0.88
*Sex (%male)*	29 (27%)	5 (36%)	24 (26%)	0.39
*Age (years)*	32 [22 – 45]	32 [22 – 45]	33 [23 – 42]	0.78
*Biopsy proven TMA*	72 (67%)	13 (93%)	59 (63%)	
***identification of mutation***	**75 (70%)**	10 (71%)	65 (70%)	0.95
*CFH*	40	6	34	
*CFI*	17	3	14	
*MCP*	10	1	9	
*C3*	11	1	10	
*CFB*			2	
*Anti-factor H Autoantibody*			2	
***Potential explanation***	**2 (13.3%)**	**1 (14.3%)**	**11 (12%)**	
*Familial form*		1	10	
*Low C3*	2	0	1	
***Idiopathic***	**21 (23.3%)**	**3 (21.4%)**	**19 (25%)**	
*Pregnancy associated HUS*	19 (18%)	1 (7%)	17 (18%)	0.151
Platelets (G/L)*	79 [48-117]	214 [186 - 242]	68 [46-100]	< 0.001
*Hb (g/dl)*	7.2 [6,6 - 9]	8.6 [6.7-10]	7.2 [6.6 - 8.8]	0.16
LDH* (UI/L)	1688 [951-2930]	664 [498-1914]	1711 [999-1953]	0.017
*Creatinine (mg/dl)*	7.3 [4.1 - 11.3]	4.3 [2.6 - 12]	7.6 [4.6 - 11.3]	0.27
*HD at the diagnosis*	13 (12%)	1 (7%)	12 (13%)	0.68
*HD during the episode*	72 (80%)	10 (71%)	62 (81.6%)	0.6
*Death Number/%*	6 (6.7%)	1 (7.1%)	5 (6.6%)	0.9

For Numeric value, data are express in Median value [25%-75%], for categories, data are express in number of observation (%). Test used, Mann Whitney test (quantitative data) and Fisher's test for proportion and rate. *Note,* Conversion factors for units, serum creatinine in mg/dL to mol/L, ×88.4; serum urea nitrogen in mg/dL to mmol/L.

To assess the severity of the episode, we evaluated the renal outcome one month after the onset of HUS. Because baseline biological data of the two cohorts seemed quite similar and in order to increase the power of the study, we pooled the two cohorts. Table 3 summarizes the outcome. 34 patients over 150 were lost-to follow up. HD was required at the acute phase in 106/150 (71%) patients. 65/150 patients (43%) remained HD-dependant. 20/150 patients (13.6%) had persistent renal failure, and only 24/150 patients (16%) had complete recovery of their renal function. No statistical difference could be highlighted between the two groups.

**Table 3:** Renal outcome three months after the last episode

	**Total**	**Patients with normal platelets count**	**Patients with thrombocytopenia**	**p**
Number of patient	150	20	130	
Death	7 (4.7%)	2 (10%)	5 (4%)	0.55
ESRD leading to chronic haemodialysis	65 (43%)	9 (45%)	56 (43%)	0.28
CKD	20 (13.6%)	4 (20%)	16 (12%)	0.33
Recovery	24 (16%)	3 (15%)	21 (16%)	0.75
Lost	34 (22%)	2 (10%)	32 (25%)	0.21

CKD, chronic kidney injury estimated GFR in ml/min/1.73 m^2^ (MDRD simplified) <60 ml/min/1.73 m^2^. Fisher's test analysis.

ESRD, End stage renal disease.

## Discussion

We report here an occurrence of 13% (20/150) of partial HUS patients defined by normal platelets count at diagnosis in two independent French cohorts. Thrombocytopenia was still absent in relapses when it was absent during the first episode. This suggests that the occurrence of partial HUS is frequent. No specific cause of partial HUS was associated with lack of thrombocytopenia.

Normal platelets count in TMA has already been described in previous study. Veyradier et al. [12] reported an occurrence of 40%. However, in this study, patients included had either HUS or TTP, with a high proportion of secondary TMA. In others clinical reports, Sellier-leclerc et al. reported an occurrence of 15% in a cohort of aHUS with complement mutation [13] and Fakhouri et al. [14] described 3 over 21 aHUS with normal platelets count in pregnancy associated HUS. In those articles details are not mentioned and little is known about platelets count evolution but the occurrence of normal platelets count seems similar to what we noticed. Recently, De Serre et al. [11] described an occurrence of 44% of patient with TMA and normal platelets count in a cohort of histological TMA. Nevertheless, some of the patients did not present with haemolytic anaemia. Consequently, we assumed that some of their description included localized TMA. In our study, patients with partial HUS reflect the whole TMA spectrum in a nephrology centre. Our study put in light that biopsy is difficult to manage in the course of HUS. Consequently, histological diagnosis of HUS is often difficult to confirm. One limitation of our study is in some patients with partial HUS, a kidney biopsy lacks to absolutely confirm the diagnosis of renal TMA. We have only indirect clinical evidence by the presence of a mutation in the genes coding for complement alternative pathway and/or response to plasmatherapy, in favour of the diagnosis of HUS.

Nevertheless, this study underlines a similar renal outcome of HUS whatever the platelets count, even if haemolysis is more severe in cases with thrombocytopenia. This similar outcome is not explained by the fact that in normal platelets count, patient did not experiment plasmatherapy. Renal outcome impacts on the prognosis of HUS. A poor outcome, leading to irreversible kidney injury or death, is described in both groups. This confirms data from De Serres et al. [11]. They described a poor renal outcome and a worse survival in the group with normal platelets count compared to the thrombocytopenic group. The authors argued that a worst outcome may be related to delay in patient care. Our patients with partial HUS had a median delay to diagnosis of 11 days. This can explain the similar outcome in our two groups. We believed that normal platelets count can delay HUS diagnosis and that kidney biopsy is the key to confirm the diagnosis. Thus we recommend performing renal biopsy if it is possible as soon as possible in patient with haemolytic anaemia and kidney impairment. It is also critical to investigate the alternative pathway of complement by measure of the complement protein and research of mutations in the genes controlling this pathway. The diagnosis could elicit the initiation of the treatment of HUS. Our data provide a strong argument that patient with partial HUS would benefit from the same therapy as for thrombocytopenic HUS.

This study questions whether platelets count is the best criterion to evaluate response to treatment in HUS. Indeed, TMA can persist even when thrombocytopenia has resolved. Improvement of kidney function or other organs involvement like brain or heart could be retained as real response criterion in HUS [15]. The LDH levels cannot be used alone as it has already been reported as a less important biomarker [16,17]. More accurate biomarkers of HUS activity are yet to be found to optimise patients’ care.

## Conclusion

Thrombocytopenia is not necessary to evoke HUS diagnosis. The association of mechanical haemolytic anaemia and renal injury in the absence of thrombocytopenia should lead to perform a renal biopsy in order to diagnose partial HUS. Normal platelets count is not associated with favourable renal prognosis in HUS.

## Abbreviations

HUS: Haemolytic and uremic syndrome; aHUS: atypical haemolytic and uremic; STEC-HUS: Shiga-toxin Escherichia coli haemolytic and uremic syndrome; TMA: Thrombotic microangiopathy; TTP: Thrombotic thrombocytopenic purpura; LDH: Lactate deshydrogenase.

## Competing interests

We declare no conflict of interest.

## Authors’ contributions

MS and KI collected and analyzed the data of the Marseille’s cohort and write the first draft of the manuscript, HVC and JMF are involved in the care of the patients of the Marseille’s cohort, FF analyzed and maintained the data of the French cohort, VFB provided the mutation screening of complement genes and maintenance of the data of the French cohort, SB designed the study, analyzed the whole dataset and write the manuscript. All authors read and approved the final manuscript.

## Pre-publication history

The pre-publication history for this paper can be accessed here:

http://www.biomedcentral.com/1471-2369/14/3/prepub
